# HER2-targeted therapies for HER2-positive early-stage breast cancer: present and future

**DOI:** 10.3389/fphar.2024.1446414

**Published:** 2024-09-16

**Authors:** Luying Xu, Yuxin Xie, Qiheng Gou, Rui Cai, Rong Bao, Yucheng Huang, Ruisi Tang

**Affiliations:** ^1^ Department of Medical Oncology, Cancer Center, West China Hospital, Sichuan University, Chengdu, China; ^2^ Breast Disease Center, West China Hospital, Sichuan University, Chengdu, China; ^3^ Department of Radiation Oncology and Department of Head & Neck Oncology, Cancer Center, West China Hospital, Sichuan University, Chengdu, China

**Keywords:** HER2-positive breast cancer, early-stage breast cancer, targeted therapy, neoadjuvant therapy, adjuvant therapy

## Abstract

Breast cancer (BC) has the second highest incidence among cancers and is the leading cause of death among women worldwide. The human epidermal growth factor receptor 2 (HER2) is overexpressed in approximately 20%–30% of BC patients. The development of HER2-targeted drugs, including monoclonal antibodies (mAbs), tyrosine kinase inhibitors (TKIs) and antibody–drug conjugates (ADCs), has improved the operation rate and pathological remission rate and reduced the risk of postoperative recurrence for HER2-positive early-stage BC (HER2+ EBC) patients. This review systematically summarizes the mechanisms, resistance, therapeutic modalities and safety of HER2-targeted drugs and helps us further understand these drugs and their use in clinical practice for patients with HER2+ EBC.

## 1 Introduction

Breast cancer (BC) is the second most common cancer and the leading cause of female mortality globally. Approximately 15%–20% of BC patients overexpress human epidermal growth factor receptor 2 (HER2) (Loibl and Gianni, 2017). Patients with HER2-positive (HER2+) BC have a more aggressive disease course, leading to a poorer prognosis than patients with other subtypes of BC (Zell et al., 2009). The development of drugs targeting the HER2 signal transduction pathway has not only greatly prolonged the survival of unresectable or metastatic patients but also significantly improved the cure rate of early-stage patients (T1–3, N0–1, M0) or locally advanced patients (T2–3, N2 or N3, M0; T4, any N, M0) (Loibl and Gianni, 2017; Gradishar et al., 2024). According to the latest recommendation, patients with BC in which HER2 immunohistochemistry (IHC) 3+ or IHC 2+/fluorescence *in situ* hybridization (FISH) results are amplified are considered to be HER2 positive and eligible for several therapies that disrupt HER2 signaling pathways (Wolff et al., 2023). The most commonly used therapy for HER2+ EBC is surgery plus HER2-targeted therapies. Trastuzumab- and pertuzumab (HP)-based regimens, such as TCbHP and THP, are the basic choices for patients receiving neoadjuvant therapy (NAT) (Gradishar et al., 2024). More than 60% of patients achieve a pathological complete response (pCR) (Breast Cancer Expert Committee of Chinese Society of Clinical Oncology, 2021). Then, if patients achieve pCR, HP is recommended for use in the adjuvant stage. For patients with non pCR, trastuzumab emtansine (T-DM1) or dual-target therapy is recommended (Burki, 2019). The 5-year disease-free survival (DFS) rates ranged from 80% to 90%. Adjuvant trastuzumab plus pertuzumab with non-anthracycline-containing or anthracycline-containing chemotherapy is considered the standard of care for postoperative patients. The 10-year breast cancer mortality was reduced by 6·4% (Bradley, 2021). Thus, adding anti-HER2 monoclonal antibodies (mAbs) to chemotherapy for HER2+ EBC is essential to reduce mortality from BC and prolong life survival. Neratinib is recommended for node-positive patients who have completed adjuvant dual-target therapy (Martin et al., 2017). Nevertheless, the systemic treatment model for HER2+ EBC is undergoing a transformation, covering various treatment stages, including adjuvant, neoadjuvant, and postneoadjuvant intensification. The treatment protocol is continually refined through diverse explorations, including novel drug combinations, immunotherapy integration, and the use of cutting-edge antibody‒drug conjugate (ADC) drugs.

Herein, this study aimed to provide a systematic summary of the mechanisms, resistance patterns, treatment options, and safety profiles of HER2-targeted medications, enhancing the current understanding of these drugs and their application in clinical settings for patients diagnosed with HER2+ EBC. A thorough search was conducted across PubMed, the Cochrane Library, Embase, and relevant oncology journals up to January 2024. Keywords such as “HER2-positive breast cancer”, “HER2-targeted therapies”, “early-stage breast cancer”, and other relevant terms were used to search for articles related to this topic.

## 2 HER2 signaling pathway

HER2 is one of the four members of the human epidermal growth factor receptor (EGFR) TKI family (Larionov, 2018) and is encoded by a gene located on the short arm q22 of chromosome 17. HER2 is made of an extracellular domain, a transmembrane domain containing two cysteine-rich repeat clusters, and an intracellular kinase domain. The extracellular domain of HER2 consists of four subdomains, numbered 1 to 4, which is the binding target of different mAbs. HER2, along with the other three EGFR family members (EGFR/ERBB1/HER1, ERBB3/HER3, and ERBB4/HER4), shares highly similar protein structures, (Slamon et al., 1987). These family members can collectively form a total of 28 combinations of homodimers or heterodimers (Wee and Wang, 2017), thereby activating their intracellular kinase domains and initiating downstream signal transduction pathways involved in cell proliferation, motility, adhesion, and resistance to apoptosis (Jiang et al., 2018).

**FIGURE 1:**
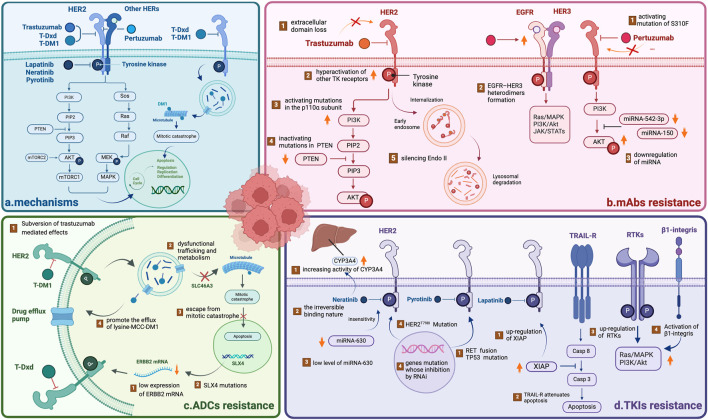
Overview of the mechanisms underlying HER2-targeted drug resistance **(A)** The mechanisms of HER2-targeted drugs, including mAbs, TKIs and ADCs. All drugs target the HER2 receptor, inhibiting the downstream PI3K/AKT and Sos/MAPK pathways. ADCs can play a cytotoxic role via the endocytosis of tumor cells, releasing free DM1 after lysosomal degradation. **(B)** The mechanism of mAb resistance. **(C)** The mechanism of ADC resistance. **(D)** The mechanism of TKI resistance. NOTE. The figure is original. Abbreviations: mAbs: monoclonal antibodies; TKIs: tyrosine kinase inhibitors; ADCs: antibody‒drug conjugates.

HER2 is unique within its family because it cannot form homodimers in a ligand-dependent manner but instead requires heterodimerization with other EGFR family proteins or spontaneous homodimerization when overexpressed (Holbro et al., 2003; Nami et al., 2018). Consequently, HER2, along with the other three members, can widely interconnect into a signaling network, leading to the overactivation of various pro-oncogenic pathways, such as the RAS-RAF-MEK-ERK-MAPK and AKT-PI3K-mTOR pathways (Wee and Wang, 2017), thereby playing a crucial role in breast oncogenesis (Tarantino et al., 2021) (Figure 1). Among the homo/heterodimers, the HER2-HER3 heterodimer is the most noteworthy. The HER2-HER3 heterodimer can fully activate all available HER2 and HER3 downstream receptors, strongly activating the PI3K-AKT pathway in addition to the RAS-ERK pathway. Many studies have focused on the link between the HER2-HER3 signaling pathway and HER2+ BC (Nami et al., 2018). Generally, the development of drugs targeting the HER2 signal transduction pathway and overcoming drug resistance are meaningful for the treatment of HER2+ BC.

## 3 Mechanisms and resistance to target medicines

### 3.1 Monoclonal antibodies

The development of mAbs targeting growth factor receptors is one of the major advances in BC treatment. These antibodies mainly target EGFR/HER and vascular endothelial growth factor (VEGF). Trastuzumab and pertuzumab are the most commonly used monoclonal drugs for treating HER2+ BC.

#### 3.1.1 Trastuzumab

**FIGURE 2:**
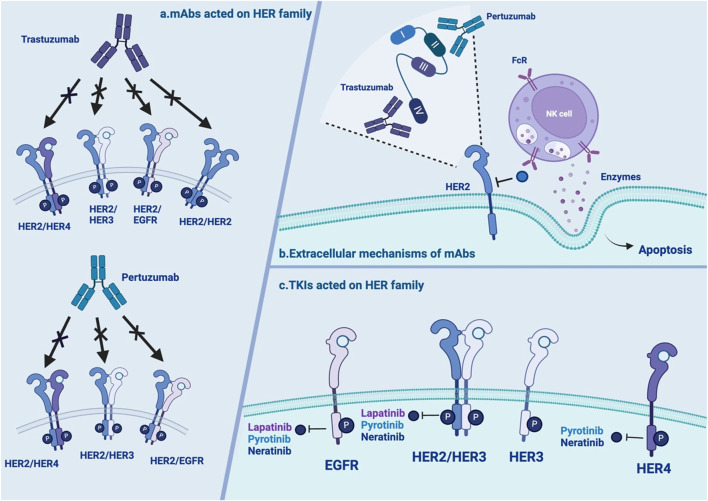
Overview of the extracellular mechanisms of mAbs and TKIs **(A)** mAbs acted on HER family: trastuzumab and pertuzumab have distinctive action on HER2 homo- and heterodimers with or without ligand. **(B)** The extracellular mechanisms of mAbs: trastuzumab and pertuzumab bind to different epitope subdomains of HER2. interact with the Fc receptors of NK cells to release ADCC. **(C)** TKIs acted on HER family: neratinib, lapatinib and pyrotinib have distinctive action on HER2 homo- and heterodimers with or without ligand. NOTE. The figure is original. Abbreviations: mAbs:monoclonal antibodies; TKI: tyrosine kinase inhibitors; EGFR: human epidermal growth factor receptor; HER2: human epidermal growth factor receptor 2; NK: natural killer; ADCC: antibody-dependent cell-mediated cytotoxicity.

Trastuzumab is a humanized recombinant monoclonal antibody (Supplementary Table S1) that has shown significant clinical benefits for treating HER2+ BC (Yip and Papa, 2021). Its mechanisms include both intracellular and extracellular mechanisms (Table 1). There are three main intracellular mechanisms involved: (1) inhibition of HER2-mediated downstream signaling pathways (Maadi et al., 2021); (2) inhibition of proteolytic cleavage of the extracellular domain of HER2 (Nami et al., 2018); and (3) reduction in proangiogenic factors (Nieto et al., 2020). Regarding the extracellular mechanism, the Fc region of trastuzumab can also bind to Fcγ receptors expressed on immune effector cells, thus inducing antibody-dependent cell-mediated cytotoxicity (Nami et al., 2018) (Figure 2). The efficacy of trastuzumab can be influenced by factors such as the level of HER2 expression on cancer cells, the presence of other mutations or alterations in signaling pathways, and the development of resistance over time (Jørgensen, 2024).

**TABLE 1:** Mechanism and resistance of drugs targeting HER2.

	Monoclonal antibody		Tyrosine kinase inhibitor			Antibody-drug conjugate	
Drugs	Trastuzumab	Pertuzumab	Neratinib	Lapatinib	Pyrotinib	T-DM1	T-DXd
Mechanism of action	(1) inhibition of HER2-mediated downstream signaling pathways; (2) inhibition of proteolytic cleavage of the extracellular domain of HER2; (3) reduction in proangiogenic factors; (4) induce antibody-dependent cell-mediated cytotoxicity by binding to the Fc receptors of immune effector cells	(1) block the constitutive activation of HER2 homologous dimers; (2) interact with the Fc receptors of natural killer cells to release antibody-dependent cell-mediated cytotoxicity	(1) competitively occupy the ATP-binding site on HER1, HER2, and HER4; (2) Alkylation or covalent bonding occurs with specific amino acid residues near the EGFR binding site	(1) binds to the ATP-binding site of the HER1/HER2 receptor’s intracellular domain	(1) alkylates cysteine residues in the intracellular ATP-binding site of pan-ErbB receptors, deactivating the receptor permanently	(1) selective delivery to HER2+ tumor cells by DM1 for trastuzumab-mediated inhibition of HER2 signaling, inhibition of HER2 extracellular domain shedding, and induction of ADCC	(1) releases cytotoxic drugs through lysosomal cathepsin cleavage after entering cells
Mechanism of resistance	(1) activating mutations in the p110α subunit of PI3K or inactivating mutations in phosphatase and tensin homolog; (2) hyperactivation of other TK receptors activating HER2 downstream signaling pathways; (3) loss of the HER2 extracellular domain; (4) silencing of the endocytic adapter protein endophilin A2	(1) S310 F disrupting the interaction between pertuzumab and HER2; (2) the formation of EGFR–HER3 heterodimers and phosphorylation of AKT and ERK1/2; (3) miRNA regulation such as downregulation of miRNA-542-3p and miRNA-150	(1) a low level of miRNA-630 induces cell insensitivity; (2) a reduction in drug target expression due to the irreversible binding nature of neratinib; (3) an increase in the activity of CYP3A4; (4) a mutation in the HER2 gatekeeper gene; and (5) a collection of genes related to neratinib resistance	(1) the upregulation of XIAP; (2) the activation of compensatory pathways mediated by the upregulation of RTKs; (3) the hyperactivation of MCL-2 or tumor necrosis TRAIL receptor; (4) the activation of β1 integrin, which promotes the activation of PI3K or the mTOR signaling axis	(1) RET gene fusion and TP53 gene mutation	(1) reduced HER2 expression, reduced T-DM1 binding, dysregulated PI3K signaling, signaling through alternative RTKs, and the tumor immune set point; (2) intratumor heterogeneity in HER2 expression and accessibility; (3) altered internalization of HER2–T-DM1 complexes; (4) impaired lysosomal release of lysine-MCC-DM1	(1) reduced target expression, epitope masking or “binding site barrier; (2) efflux pump, internalization defect or lysosome processing; (3) high expression of ERBB2 mRNA; (4) recurrent mutations in the SLX4 gene
Percentage of resistance	Up to 30% of patients with HER2-positive EBC do not achieve a complete response (Lavaud and Andre, 2014; Stocker et al., 2016)	-	Approximately 37% patients at all neratinib doses have no objective response after using HP treatment (Guo et al., 2023)	Approximately 50%–60% of patients with HER2-positive EBC develop resistance to lapatinib (Wahdan-Alaswad et al., 2020)	-	Acquired resistance: 20% (Barok et al., 2014); primary resistance: more infrequent than acquired	-

Abbreviations: HER2: human epidermal growth factor receptor 2; EBC: early-stage breast cancer; PI3K: phosphoinositide 3-kinase; EGFR: human epidermal growth factor receptor; ATP, adenosine triphosphate; XIAP: X inhibitor of apoptosis protein; RTKs: receptor tyrosine kinases; MCL-2: mantle cell lymphoma; TRAIL: tumor necrosis factor-related apoptosis-inducing ligand; mTOR: mammalian target of rapamycin; T-DM1: trastuzumab emtansine.

Trastuzumab resistance can be categorized as follows: 1. Intrinsic resistance occurs when disease lesions develop within 12 months after starting adjuvant trastuzumab treatment. 2. Adaptive resistance is observed in patients with EBC who have local recurrence and metastasis during (neo)adjuvant trastuzumab treatment or within 12 months after the end of trastuzumab treatment (Wong et al., 2011). Clinical results revealed that one-third of patients respond to trastuzumab but develop resistance to this drug (Bartsch et al.). In the HERA trial, researchers discovered that after a median follow-up of 11 years, compared with the observation group, 1 year of trastuzumab treatment significantly reduced the risk of disease recurrence or death, but 2 years of treatment with trastuzumab did not improve long-term disease-free survival (DFS) (Cameron et al., 2017). Several mechanisms may contribute to resistance: (1) activating mutations in the p110α subunit of PI3K or inactivating mutations in phosphatase and tensin homolog (PTEN); (2) hyperactivation of other TK receptors activating HER2 downstream signaling pathways (Maadi et al., 2021); (3) loss of the HER2 extracellular domain (Derakhshani et al., 2020); and (4) silencing of the endocytic adapter protein endophilin A2, which causes defects in HER2 internalization (Derakhshani et al., 2020) (Table 1; Figure 1).

#### 3.1.2 Pertuzumab

Pertuzumab is another widely used monoclonal antibody (Supplementary Table S1). In contrast to trastuzumab, which binds to epitope subdomain 4, pertuzumab specifically binds to epitope subdomain 2 of the HER2 receptor (Mercogliano et al., 2023). This can block the constitutive activation of HER2 homologous dimers and prevent HER2 from forming heterodimers with other HER receptors, especially HER3 (Sendur et al., 2012). Pertuzumab can also interact with the Fc receptors of natural killer (NK) cells to release antibody-dependent cell-mediated cytotoxicity (ADCC) (Mercogliano et al., 2023) (Table 1; Figure 2). Due to different binding targets, pertuzumab combined with trastuzumab is more effective in treating HER2+ BC (Fazal et al., 2023; Vieira et al., 2023). In fact, the combination of two monoantibodies and taxanes has been the first-line therapy for HER2+ BC. The curative effect is comparable whether taxane is added or not (Woodward et al., 2019; Miles et al., 2021). Several studies have reported resistance to pertuzumab, suggesting mechanisms linked to (1) S310F, an activating mutation in the HER2 extracellular domain, disrupting the interaction between pertuzumab and HER2 (Zhang et al., 2019); (2) the formation of EGFR–HER3 heterodimers and phosphorylation of AKT and ERK1/2 (Hutcheson et al., 2007); and (3) miRNA regulation, such as downregulation of miRNA-542-3p (Ma et al., 2015) and miRNA-150 (Wuerkenbieke et al., 2015) (Table 1; Figure 1).

### 3.2 Tyrosine kinase inhibitors (TKIs)

Up to 30%–50% of BC patients develop brain metastasis (BM), which is an important event influencing their survival and quality of life (Brosnan and Anders, 2018). In the treatment of BM, mAbs may have trouble crossing the blood‒brain barrier (BBB), whereas small molecule TKIs fill the drug treatment gap. EGFR-TKI inhibitors competitively bind to the intracellular adenosine triphosphate (ATP)-binding domain of EGFR, leading to the inhibition of TK phosphorylation and subsequent blockade of downstream signaling (Figure 2). This activity could effectively promote cancer cell apoptosis and prevent proliferation (Traxler, 2003).

#### 3.2.1 Neratinib

Neratinib is an irreversible chloroanilino-quinazoline inhibitor of HER2 (Tsou et al., 2005). It targets EGFR (HER1) and HER4. In preclinical *in vitro* models, neratinib effectively and selectively inhibited the proliferation of HER2+ BC cell lines (Xuhong et al., 2019). It reduced the autophosphorylation of the HER2 receptor, which eventually led to the inhibition of downstream MAPK and AKT phosphorylation (Rabindran et al., 2004) (Table 1).

Approximately 70% of patients with HER2+ BC develop innate or acquired resistance to HER2-targeted agents, and neratinib is no exception (Arribas et al., 2011). Interestingly, neratinib-resistant cells are cross-resistant to all other HER2-targeted drugs, and this resistance is bidirectional. The following mechanisms of resistance may explain this issue: (1) a low level of miRNA-630 induces cell insensitivity (Corcoran et al., 2014); (2) a reduction in drug target expression due to the irreversible binding nature of neratinib (Azuma et al., 2014); (3) an increase in the activity of CYP3A4 (Breslin et al., 2017); (4) a mutation in the HER2 gatekeeper gene (Hanker et al., 2017); and (5) a collection of genes related to neratinib resistance (Seyhan et al., 2012) (Table 1; Figure 1).

#### 3.2.2 Lapatinib

Lapatinib is a reversible, selective, oral dual TKI that binds to the ATP-binding site of the HER1/HER2 receptor’s intracellular domain (Wahdan-Alaswad et al., 2020) (Table 1). Lapatinib is often used in high-risk HER2+ MBC patients who develop resistance to trastuzumab (Guarneri et al., 2012). The European Union (EU) has authorized the combination of lapatinib plus trastuzumab for hormone receptor (HR)+/HER2+ MBC patients who have previously received trastuzumab (Singh et al., 2022). It has also been approved for postmenopausal HER2+ MBC patients (Khan et al., 2020). However, the use of lapatinib in HER2+ EBC patients is still being explored.

Many potential mechanisms of resistance to lapatinib have been reported, including (1) the upregulation of X inhibitor of apoptosis protein (XIAP), which can change the cell death cascade (Eustace et al., 2018); (2) the activation of compensatory pathways mediated by the upregulation of receptor tyrosine kinases (RTKs) (Goyette et al., 2018); (3) the hyperactivation of mantle cell lymphoma (MCL-2) or tumor necrosis factor-related apoptosis-inducing ligand (TRAIL) receptor (Aird et al., 2010; Eustace et al., 2018); and (4) the activation of β1 integrin, which promotes the activation of phosphoinositide 3-kinase (PI3K) or the mammalian target of rapamycin (mTOR) signaling axis (Junttila et al., 2009; Toomey et al., 2017) (Table 1; Figure 1).

#### 3.2.3 Pyrotinib

Pyrotinib is a new generation of anti-HER2 drugs made in China. In 2018, the drug received conditional approval from the Chinese State Drug Administration for use in combination with capecitabine to treat patients with HER2+ MBC (Blair, 2018). Recently, it has been shown to be effective for neoadjuvant therapy (NAT) (Wu et al.,; Wang et al., 2023). Pyrotinib is an irreversible TKI that acts on pan-ErbB receptors (Li et al., 2017). By covalently binding to ATP-binding sites in HER intracellular kinase domains, it inhibits the autophosphorylation of the HER dimer and blocks the Ras/Raf/MEK/MAPK and PI3K/Akt signaling pathways, thereby preventing tumor progression (Li et al., 2017; Ma et al., 2017) (Table 1).

Few cases of resistance have been reported. However, a case reported that there might be rapid progression after drug resistance. Researchers have speculated that rapid progression after pyrotinib resistance may be due to RET gene fusion and TP53 gene mutation. However, the underlying mechanism remains to be investigated (Jiang et al., 2023). Regarding resistance to pyrotinib, a previous study revealed that the P110α inhibitor alpelisib could exert synergistic effects with pyrotinib, reversing resistance in HER2+ BC and providing ideas for overcoming resistance (Chen et al., 2023) (Table 1; Figure 1).

### 3.3 Antibody‒drug conjugates

ADCs are a new class of protein-based therapeutic drugs that combine the targeting ability, high selectivity, and stability of mAbs with the anticancer potential of high-efficiency payoffs. The application of ADCs can increase precision drug delivery in cancer cells while protecting healthy tissues and cells from chemotherapeutic damage (Abbas et al., 2021). Each ADC contains three parts: a monoclonal antibody, linker, and cytotoxic drug. There are two types of linkers: cleavable and noncleavable. Both are used in ADCs that have been developed in clinical trials or are currently being developed (Chari et al., 2014). There are two types of cytotoxic drugs: microtubule inhibitors and DNA-damaging agents (Moldenhauer et al., 2012). Compared to standard chemotherapeutic drugs, the cytotoxic compounds used in the ADC structure can confer greater cell killing power to mAbs (Chari, 2008).

#### 3.3.1 Trastuzumab emtansine

Trastuzumab emtansine (T-DM1) is an ADC approved by the Food and Drug Administration (FDA) that consists of the maytansinoid toxin DM1 linked to the humanized monoclonal antibody trastuzumab via a thioether-based chemical linker (Lewis Phillips et al., 2008). In T-DM1, the monoclonal component is directed toward HER2, which allows the delivery of the cytotoxic drug through receptor-mediated endocytosis in a selective way, reducing the toxicity to the off-target tissue (Chari, 2016; Birrer et al., 2019). T-DM1 has diverse mechanisms: selective delivery to HER2+ tumor cells by DM1 for trastuzumab-mediated inhibition of HER2 signaling, inhibition of HER2 extracellular domain shedding, and induction of ADCC (Hunter et al., 2020) (Table 1).

Numerous mechanisms may be responsible for T-DM1 resistance: (1) reduced HER2 expression, reduced T-DM1 binding, dysregulated PI3K signaling, signaling through alternative RTKs, and the tumor immune set point; (2) intratumor heterogeneity in HER2 expression and accessibility; (3) altered internalization of HER2–T-DM1 complexes; and (4) impaired lysosomal release of lysine-MCC-DM1(Hunter et al., 2020) (Table 1; Figure 1).

#### 3.3.2 Trastuzumab deruxtecan

Trastuzumab deruxtecan (T-DXd) is an efficient ADC formed by the addition of topoisomerase agent I and a cleavable tetrapeptide-based linker to trastuzumab (Modi et al., 2020). The conjugate is stable in plasma and releases cytotoxic drugs through lysosomal cathepsin cleavage after entering cells (Xu et al., 2019) (Table 1). Compared to T-DM1, T-DXd has a greater drug-to-antibody ratio with a favorable pharmacokinetic profile (Doi et al., 2017).

Currently known resistance mechanisms are either “monoclonal” (i.e., reduced target expression, epitope masking, “binding site barrier”) or “payload” (i.e., efflux pump, internalization defect/lysosome processing) (García-Alonso et al., 2020; Drago et al., 2021; Nessler et al., 2021). ERBB2 mRNA expression has been shown to be positively correlated with progression-free survival (PFS) and overall survival (OS) after T-DM1 treatment (Brasó-Maristany et al., 2023). In the DAISY trial, genomic analysis of patients revealed that approximately 20% of patients with T-DXd progression had recurrent mutations in the SLX4 gene (Andre et al., 2022). These findings indicate that the expression and mutation of some genes can affect the resistance of T-DXd. More preclinical and translational studies should be conducted in conjunction with ongoing clinical trials to gain insight into T-DXd resistance mechanisms (Table 1; Figure 1).

## 4 Therapeutic modalities and safety of neoadjuvant therapy

NAT is a systemic preoperative therapy that is suitable for patients with locally advanced BC (Agostinetto et al., 2022; Wang Q. et al., 2022). It can reduce or eliminate metastatic lymph node cells and cancer lesions to reduce the postoperative recurrence rate (Spring et al., 2019; Spring et al., 2022). NAT can even enable some inoperable patients to undergo surgery (Supplementary Table S2).

### 4.1 Single HER2-directed therapy

In 2004, the first introduction of neoadjuvant chemotherapy (NACT) with trastuzumab in a phase III randomized clinical trial (RCT) demonstrated the significant effects of trastuzumab in neoadjuvant therapy. More studies have further confirmed these findings (Buzdar et al., 2005; Untch et al., 2011; Gianni et al., 2014).

#### 4.1.1 Trastuzumab combined with docetaxel

BC is sensitive to some chemotherapeutic agents, such as anthracyclines or taxanes (Pritchard et al., 2008; Di Leo et al., 2011). Considering that anthracyclines plus trastuzumab are associated with cardiotoxicity (Dempsey et al., 2021), trials on the combination of docetaxel and trastuzumab are underway.

In a single-arm trial of NSABP-B27 plus trastuzumab, 121 high-risk HER2+ BC patients were included. After standard treatment, as reported for NSABP-B27, trastuzumab was given for 1 year. Following NAT, 119 patients underwent surgery, of whom 59 (49.6%) achieved pCR. Cardiotoxicity was not reported (Abdel-Razeq et al., 2018). Another trial evaluated trastuzumab in addition to docetaxel and adriamycin. In a phase II trial, 50 patients with stage II or III HER2+ BC were recruited. Six cycles of pegylated liposomal doxorubicin (PLD) plus docetaxel and trastuzumab were given. The total pathologic complete response (tpCR) rate and objective response rate (ORR) were 48.0% (95% CI, 33.7%–62.6%) and 84.0% (95% CI, 70.9%–92.8%), respectively (Wang H. et al., 2022). These trials confirmed the superior performance of trastuzumab plus docetaxel. Notably, trastuzumab has several adverse effects, especially cardiotoxicity. Some drugs, such as lisinopril, carvedilol, and β-escin, may help reduce toxicity to minimize interruptions (Guglin et al., 2019; Park et al., 2022). Trastuzumab is also associated with severe thrombocytopenia. Chemotherapy with pertuzumab may be an alternative treatment option (Huff et al., 2022).

#### 4.1.2 Trastuzumab combined with paclitaxel (albumin paclitaxel)

Paclitaxel is the first drug found to interact with tubulin aggregates, binding tightly to microtubules and stabilizing them. Trastuzumab plus paclitaxel could increase the therapeutic effect in HER2+ BC (Pegram et al., 2000; Ding et al., 2017; Wang et al., 2017; Zhang et al., 2020).

The TECHNO trial included 217 HER2+ BC patients (with a tumor size ≥2 cm or inflammatory BC). The patients received four 3-week cycles of epirubicin plus cyclophosphamide followed by four 3-week cycles of paclitaxel plus trastuzumab before surgery. Complete treatment with trastuzumab was continued 1 year after surgery. The 3-year DFS (88% vs 73%, *P* = 0.1) and OS (96% vs 86%, *P* = 0.25) were significantly improved in patients who achieved a pCR. Cardia toxicity was observed in 8 patients (3.7%) (Untch et al., 2011). Similar pCR results were also obtained when paclitaxel was combined with trastuzumab plus carboplatin (Ding et al., 2017; Wang et al., 2017; Waks et al., 2022). In recent years, nanoparticle albumin-bound paclitaxel (nab-PTX) has been developed. A multicenter phase II trial enrolled 29 HER2+ operable BC patients. Each patient received four cycles of nab-PTX with trastuzumab, followed by four cycles of 5-fluorouracil/epirubicin/cyclophosphamide (FEC) every 3 weeks. The pCR rate was 74.0%. The most frequent toxicity was sensory neuropathy (96.6%) (Tokunaga et al., 2019). Similarly, a phase II trial of neoadjuvant nab-PTX plus trastuzumab was conducted. Nab-PTX plus trastuzumab was given every 3 weeks for four cycles to evaluate its efficacy in terms of the pCR rate for small (≤3 cm), node-negative, pure HER2 BC. Among the 18 patients, 66.7% achieved pCR. The incidence of severe adverse events (AEs) is quite low (Tanaka et al., 2019). Using nanoalbumin as a carrier can reduce the toxicity of paclitaxel and improve targeting accuracy. Compared with traditional drugs, nanocarrier composite drugs show great advantages and have broader therapeutic prospects.

#### 4.1.3 Trastuzumab deruxtecan (T-DXd)

A phase III multicenter trial concluded that the efficacy and safety of T-DXd were better than those of T-DM1 in HER2+ MBC patients previously treated with trastuzumab and taxane (Cortés et al., 2022). Based on the promising results of T-DXd in the advanced/metastatic setting, numerous clinical trials are currently underway to investigate the effectiveness of T-DXd in NAT for HER2+ BC. Xu et al. performed a reanalysis based on clinical trials. They strengthened the conclusion that in NAT strategies, T-DXd plays an active role in treating HER2+ cancers, especially breast carcinoma (Xu et al., 2022).

Neoadjuvant T-DXd is being evaluated in the DESTINY-Breast11 trial for locally advanced or inflammatory HER2+ BC. This trial compared T-DXd monotherapy with T-DXd followed by paclitaxel and trastuzumab plus pertuzumab (THP) or doxorubicin and cyclophosphamide (ddDC) followed by THP. The SHAMROCK study will investigate neoadjuvant T-DXd in HER2+ EBC patients. Researchers hypothesize that this novel adaptive trial design will achieve high pCR rates and avoid unnecessary toxicity while also exploring predictive and prognostic biomarkers for treatment (Dowling et al., 2024). Since the DESTINY-Breast04 trial revealed that T-DXd showed marked effectiveness in patients with HER2-low MBC, relevant trials in the neoadjuvant field are ongoing (Modi et al., 2022; von Arx et al., 2023). T-DXd has several common adverse effects, such as nausea (73%), vomiting (38%), hair loss (37%), fatigue (36%) and diarrhea (27%) (André et al., 2023). Approximately 15.4% of patients with different solid tumors develop interstitial lung disease (ILD) upon T-DXd treatment, which is considered to be a specific AE (Powell et al., 2022).

Apparently, T-DXd is a promising newcomer in the field of NAT for BC, but there are still numerous challenges that require further research. For example, what is the optimal sequence for administering T-DXd in combination with other medications?

### 4.2 Dual HER2-directed therapy

#### 4.2.1 Trastuzumab combined with pertuzumab

As mentioned earlier, dual blockade of HER2 activation and downstream signaling was achieved through the combination of trastuzumab and pertuzumab by binding to different epitopes of the HER2 receptor. Trastuzumab inhibits HER2 dimerization, whereas pertuzumab impedes HER2 heterodimerization with other HER family receptors, especially HER3. Additionally, both agents can also induce ADCC, thus enhancing the antitumor effect. Furthermore, combination therapy may reduce the occurrence of drug resistance.

To date, the preferred treatment for NATs is trastuzumab plus pertuzumab. An international multicenter phase III KRISTINE trial was conducted to evaluate whether targeted therapy could replace conventional chemotherapy via NAT for HER2+ BC patients. The study included 444 stage II-III operable HER2+ BC patients who were randomly assigned to NAT with T-DM1 plus pertuzumab (T-DM1+P) or docetaxel, carboplatin, or trastuzumab plus pertuzumab (TCbHP). The results confirmed that the pCR rate of TCbHP was 11.3% greater (Hurvitz et al., 2018). Currently, TCbHP has become the first-line NAT for HER2+ BC. Furthermore, many studies have shown that adding other drugs to this combination can provide additional benefits. In the NeoSphere trial, pertuzumab and trastuzumab plus docetaxel significantly improved the pCR rate (45.8%) compared with trastuzumab plus docetaxel (29.0%), pertuzumab plus docetaxel (24.0%), or pertuzumab plus trastuzumab (16.8%) (Gianni et al., 2012). Patients given neoadjuvant trastuzumab, pertuzumab and docetaxel further showed significantly improved 5-year PFS and DFS (Gianni et al., 2016; Huang et al., 2024). Additionally, neoadjuvant dual blockade with trastuzumab and pertuzumab plus paclitaxel achieved a pCR rate of 90.5% in the WSG-ADAPT phase II trial (Nitz et al., 2017).

Many trials using dual anti-HER2 therapy in the NAT have shown pCR rates of approximately 60% or greater, while cardiaotoxicity is rare. A BERENICE trial focusing on cardiac safety showed that the incidence of cardiac side effects was low regardless of anthracycline use (Dang et al., 2022). In this trial, there were no new cardiac issues and a low incidence of Class III/IV heart failure (only one patient). A systematic review and meta-analysis of randomized controlled trials analyzed the safety of pertuzumab combined with trastuzumab compared to trastuzumab alone for the treatment of HER2+ BC. Compared to those associated with monotherapy, grade 3 or higher febrile neutropenia, diarrhea, and anemia as well as heart failure were more frequently reported with dual therapy. However, no significant difference in serious adverse effects (AEs) was observed between the two groups (Liu X. et al., 2022). Similarly, Lynce reported no significant difference in the incidence of adverse cardiac events between pertuzumab plus trastuzumab and trastuzumab alone (Lynce et al., 2019). All these trials showed the advantages of dual anti-HER2 therapy, which improved the treatment effect without obviously increasing AEs. This is probably due to the reduced use of conventional chemotherapy (Pérez-García et al., 2021; van der Voort et al., 2021). According to a multicenter Turkish oncology study group, NAT with docetaxel, trastuzumab and pertuzumab was associated with a significantly increased incidence of side effects such as anemia, nausea, vomiting, myalgia, alopecia, and mucositis (Özdemir et al., 2022).

Overall, trastuzumab combined with pertuzumab leads to better outcomes for early-stage patients with fewer resistance events and fewer serious adverse events, making it one of the best options for HER2+ BC therapy. In addition, how dual-target therapy can be combined with other drugs, such as docetaxel and paclitaxel, to obtain better efficacy needs further exploration.

#### 4.2.2 Trastuzumab combined with TKI

##### 4.2.2.1 Pyrotinib

RCTs and retrospective real-world studies have shown the efficacy of pyrotinib in HER2+ MBC patients (Anwar et al., 2021; Li et al., 2021; Xu et al., 2021; Yan et al., 2022). The use of NATs in combination with pyrotinib for the treatment of HER2+ BC is being discussed.

In the PHEDRA trial, 355 patients were assigned randomly to receive pyrotinib or placebo in combination with trastuzumab and docetaxel before surgery; the pyrotinib group had significantly greater rates of tpCR (41.0% vs 22.0%) and breast pCR (43.8% vs 23.7%) than did the placebo group (Wu et al., 2022). In a phase II trial, 69 HER2+ BC patients were subjected to NAT involving docetaxel, carboplatin, trastuzumab, and pyrotinib (TCbHy), resulting in a pCR rate of 55.1% (Liu Z. et al., 2022). Similarly, in the phase II NeoATP trial, 53 patients with HER2+ local advanced BC (stage IIA–IIIC) achieved a pCR rate of 69.81% after undergoing NAT with pyrotinib plus trastuzumab and paclitaxel-cisplatin (Yin et al., 2022). These results demonstrated that pyrotinib could significantly improve the pCR rate and ORR of patients receiving NAT. We look forward to observing a significantly greater pCR rate with the use of pyrotinib, which is associated with significantly improved DFS and OS. Moreover, further exploration is needed to enhance the effectiveness of chemotherapy combinations and manage the balance between the efficacy and toxicity of new therapeutic regimens.

#### 4.2.3 Lapatinib

Lapatinib is mainly used as a first-line treatment for trastuzumab-resistant patients in China. In subsequent trials, dual-targeted combination therapy with trastuzumab and lapatinib significantly improved the efficacy of overlapatinib alone (Yuan et al., 2022). The NeoALTTO trial assigned 455 patients to receive trastuzumab plus lapatinib, lapatinib alone, or trastuzumab alone, and a pCR was achieved in 51.3%, 24.7%, and 29.5%, respectively, which demonstrated the superior efficacy of trastuzumab plus TKI in the neoadjuvant setting (Baselga et al., 2012). In the Neo-LaTH trial, 212 patients were randomized to receive different durations of neoadjuvant anti-HER2 therapy with trastuzumab plus lapatinib followed by weekly paclitaxel. After surgery, the patients were treated with trastuzumab for 1 year. The results showed that the 5-year DFS and OS rates were 87.8% and 95.6%, respectively (Tokunaga et al., 2021). A meta-analysis incorporating 1,410 patients revealed that, compared with trastuzumab monotherapy, lapatinib plus trastuzumab improved recurrence-free survival significantly (HR = 0.62, 95% CI 0.46–0.85) and OS (HR = 0.65, 95% CI 0.43–0.98) upon combination with NACT (Guarneri et al., 2022). These findings suggest that the combination of trastuzumab with TKIs may represent a promising neoadjuvant treatment approach. Additionally, dual HER2 blockade can lead to a decrease in the duration of chemotherapy. However, further trials are needed to determine how much shorter the duration of chemotherapy is.

#### 4.2.4 Dual HER2-directed therapy combined with immunotherapy

For patients at high risk of HER2+ EBC, TCbHP is recommended as one of the first options for NAT and can significantly improve the pCR of patients.

At the 2023 SAN Antonio Breast Cancer Seminar (SABCS), Luca Gianni introduced the PD-L1 inhibitor atezolizumab to HP NATs, thus verifying the feasibility of improving the efficacy of NATs for treating HER2+ BC by modulating the immune system (Gianni et al.). In the APTneo Michelangelo trial, 661 patients with HER2+, early high-risk and locally advanced BC were divided into three groups: A, B1 and B2. Group A received TCbHP (6 cycles); group B1 received doxorubicin and cyclophosphamide (DC) (3 cycles), followed by TCbHP (3 cycles), and atezolizumab was also used as immunotherapy during these 6 cycles; group B2 received the same treatment as group A while receiving atezolizumab. Exploratory analyses revealed that compared with the combination of TCbHP and atezolizumab, TCbHP combined with atezolizumab plus DC significantly increased the pCR rate by 9.9%. Possible mechanisms contributing to this result are the effect of anthracycline chemotherapy agents or the enhancement of DC by atezolizumab. This conclusion is worthy of further verification and exploration. In addition, good tolerability is a bright spot of this treatment therapy, providing a positive signal for future studies related to neoadjuvant immunotherapy for BC.

## 5 Therapeutic modalities and safety of adjuvant therapy

Adjuvant therapy is given after surgery to clear any remaining cancer cells in the body. The advantage of adjuvant therapy is that through the study of postoperative tumor tissue, patients can choose personalized therapy to achieve the best treatment results (Supplementary Table S3).

### 5.1 Single HER2-targeted therapy

#### 5.1.1 Trastuzumab

The administration of adjuvant trastuzumab plus chemotherapy for 1 year became the standard of care for patients with HER2+ EBC according to the results of four major adjuvant trials (NCCTG N9831, NSABP B-31, HERA and BCIRG-006) (Piccart-Gebhart et al., 2005; Romond et al., 2005; Slamon et al., 2009). The NSABP B-31/NCCTG N9831 studies demonstrated that anthracycline combined with cyclophosphamide followed by paclitaxel combined with trastuzumab (AC-TH) was superior to conventional anthracycline combined with cyclophosphamide followed by paclitaxel (AC-T) (Romond et al., 2005). BCIRG 006 proved that docetaxel and carboplatin combined with trastuzumab (TCbH) were superior to AC-T and could be used as alternative adjuvant therapies. The study showed that the long-term efficacy of TCbH and AC-TH were similar after 10 years of long-term follow-up, and the incidence of cardiac dysfunction in patients treated with TCbH was low (Slamon et al., 2009). Therefore, for patients with higher cardiac safety requirements, TCbH can be selected. In addition, the study showed that the 5-year recurrence and metastasis risk of HER2+ and T1abN0M0 patients was 5 times greater than that of HER2-low patients, suggesting that small-tumor, lymph node-negative, HER2+ BC patients still had a greater recurrence risk than small-tumor, HER2-low BC patients. However, for such patients, chemotherapy can be further reduced based on trastuzumab. Previous studies have suggested that the 2-year DFS and OS rates of EBC patients treated with TCbH are 97.8% and 99.2%, respectively (Jones et al., 2013). The APT study suggested that for patients with small tumors (≤3 cm) and HER2+ BC treated with weekly paclitaxel and trastuzumab followed by trastuzumab every 3 weeks (wTH), the 3-year DFS rate reached 98.7% (Tolaney et al., 2019). Therefore, for low-risk, T1N0, and HER2+ BC patients, TCH or wTH can be considered.

#### 5.1.2 Neratinib

In a phase III trial, 2840 HER2+ EBC patients who completed adjuvant chemotherapy plus trastuzumab were enrolled. The patients were randomly assigned to receive neratinib (n = 1,420) or placebo (n = 1,420) for 1 year based on hormone receptor and lymph node status. After a median follow-up of 5.2 years, the neratinib group had fewer invasive disease-free survival (iDFS) events than did the placebo group (95% CI 0.57–0.92, *p* = 0.0083). The 5-year iDFS for patients receiving neratinib was 90.2% versus 86.7% for those receiving placebo. The most common grade 3–4 AEs in the neratinib group compared to those in the placebo group were diarrhea, vomiting, and nausea. Compared with placebo, there was no evidence that neratinib increased the risk of long-term toxicity or diarrhea associated with long-term adverse outcomes (Martin et al., 2017). Similar AEs were reported in a 60-participant trial. Gastrointestinal disorders (57%), including diarrhea (42%), nausea (28%), and vomiting (13%), were the most common. It can be controlled with antidiarrheal agents and dose adjustments (Echavarria et al., 2017). These studies further confirmed the value of neratinib in adjuvant therapy based on trastuzumab therapy. In 2017, the FDA approved neratinib for extended adjuvant treatment of HER2+EBC (Hug et al., 2010).

#### 5.1.3 Trastuzumab emtansine

Patients with residual invasive lesions after NACT combined with anti-HER2-targeted therapy for HER2+ EBC have a greater risk of disease recurrence and death. The KATHERINE study included HER2+ EBC patients with residual invasive disease in the breast/axilla at surgery after receiving NAT containing taxane and trastuzumab. Patients were randomly assigned to receive adjuvant T-DM1 or trastuzumab for 14 cycles. The initial analysis of the trial, reported in the 2018 SABCS, revealed a significant increase of 11.3% in 3-year DFS in the T-DM1 group compared to the trastuzumab group(von Minckwitz et al.). The risk of invasive recurrence or death was reduced by 50%. In 2023, SABCS published OS data from the KATHERINE trial (Loibl et al., 2023; von Minckwitz et al.). After a median follow-up of 8.4 years, intensive T-DM1 adjuvant therapy significantly improved the OS of HER2+ EBC patients who still had invasive lesions after NAT (HR = 0.66, 95% CI 0.51–0.87; *P* = 0.0027). The 7-year OS rates of patients receiving T-DM1 and trastuzumab were 89.1% and 84.4%, respectively, with a difference of 4.7%. In the intent to treat (ITT) population, the iDFS benefit of T-DM1 persisted at long-term follow-up (HR = 0.54, 95% CI 0.44–0.66). The 7-year iDFS rates of patients receiving T-DM1 and trastuzumab were 80.8% and 67.1%, respectively. In addition, no new safety concerns arose with the extended follow-up.

The results changed the standard treatment concept for HER2+ EBC. T-DM1 became the standard intensive adjuvant treatment for HER2+ EBC patients who did not achieve pCR (nonpCR) after NAT.

Since the KATHERINE trial, other studies have explored similar topics in China (Huang et al.). After surgery following NACT, HER2+ EBC patients and those with residual aggressive disease were randomly assigned to receive adjuvant T-DM1 or trastuzumab. Compared with trastuzumab, T-DM1 treatment resulted in a 43% reduction in the risk of iDFS events, with similar results for secondary endpoints. As in the global population, Chinese patients treated with T-DM1 had more grade ≥3 AEs and adverse reactions leading to discontinuation than patients treated with trastuzumab. The most common AE (≥3 grade) of T-DM1 was thrombocytopenia (21.6%), which occurred more frequently than in the global population (5.7%). Consistent with the findings in the global study population, compared with trastuzumab, T-DM1 markedly decreased the risk of recurrence or death in Chinese patients. In addition, thrombocytopenia, elevated liver transaminases, and peripheral neuropathy are common toxicities of T-DM1 therapy (Wolska-Washer and Robak, 2019).

#### 5.1.4 Trastuzumab deruxtecan

Based on a series of studies, such as DS8201-A-J101, DESTINY-Breast01, and DESTINY-Breast02, T-DXd has successfully become an important treatment for BC (Ogitani et al., 2016; André et al., 2023; Saura et al., 2024). In the DESTINY-Breast03 study, T-DXd outperformed T-DM1 with better results, making T-DXd the gold standard for second-line treatment of HER2+ BC (Hurvitz et al., 2023). However, the pace of anti-HER2 treatment with T-DXd continues. What is the effect of T-DXd as a first-line treatment? Does the drug yield better results in adjuvant therapy? These issues need to be explored in more clinical studies. An international multicenter phase III trial, DESTINY-Breast05, is exploring T-DXd as an adjunct to intensive therapy (Geyer et al., 2021). In this study, high-risk HER2+ BC patients who did not achieve pCR after NAT were randomized to receive 14 cycles of T-DXd or T-DM1. The primary endpoint was iDFS, and the secondary endpoint included DFS. This study will further challenge the standard of intensive T-DM1 adjuvant therapy proposed by KATHERINE’s study, and whether T-DXd can rewrite the early treatment landscape of HER2+ BC is unknown.

### 5.2 Dual HER2-directed therapy

The multinational randomized APHINITY trial (NCT01358877, BIG4–11/BO25126/TOC4939G) showed that the addition of pertuzumab to trastuzumab plus standard chemotherapy as adjuvant therapy significantly improved iDFS in patients with HER2+ EBC (von Minckwitz et al., 2017). In the APHINITY trial, a total of 4,805 patients were randomly assigned to receive chemotherapy and trastuzumab plus either pertuzumab or placebo. The 6-year rate of iDFS was 91% in the pertuzumab group and 88% in the placebo group, with a hazard ratio for an invasive disease event of 0.76 in favor of pertuzumab (Piccart et al., 2021). In view of the superiority of dual-target therapy, the first-line treatment recommended by the National Comprehensive Cancer Network (NCCN) guidelines is still trastuzumab and pertuzumab combined with chemotherapy.

When using ADCs, dual-target blocking by the addition of TKIs may improve therapeutic efficacy. The combined ADC and TKI strategy was demonstrated in the NAT in the TEAL study, with better outcomes compared to standard therapy (paclitaxel, trastuzumab, and pertuzumab combination) (Patel et al., 2019). However, at present, there is no relevant research on the combination of ADC and TKI drugs for adjuvant therapy.

In summary, with the advancement of new drugs, a wider range of targeted therapies is now accessible to both doctors and patients. However, the accurate selection of personalized medicine and the optimization of combination therapies remain crucial issues that warrant attention. The potential combination of ADCs and TKIs in adjuvant therapy is a key consideration, necessitating further trials to validate their efficacy. It is essential to conduct risk stratification among different patient groups to determine the optimal duration of neoadjuvant/adjuvant therapy, thereby avoiding unnecessary treatments and minimizing side effects such as cardiotoxicity and diarrhea.

## 6 Discussion

The therapeutic landscape for HER2+ BC has been significantly transformed by the advent of targeted therapies, including mAbs, TKIs, and ADCs. Each class of drugs offers unique mechanisms of action and has distinct advantages and limitations. They are integral in both adjuvant therapy, enhancing postsurgical outcomes, and neoadjuvant therapy, improving the postsurgical tumor response. Ongoing research continues to refine their application, aiming to optimize patient-specific treatment strategies and combat resistance.

mAbs, such as trastuzumab and pertuzumab, have revolutionized the treatment of HER2+ BC by specifically targeting the HER2 receptor. Their high specificity and ability to induce ADCC make them potent agents in the fight against cancer. However, the development of resistance and the potential for immune-related adverse events are challenges that need to be addressed. The combination of mAbs, such as trastuzumab and pertuzumab, has shown synergistic effects in clinical trials, leading to improved pCR and DFS.

TKIs, such as neratinib and lapatinib, disrupt the intracellular signaling pathways activated by HER2, thereby inhibiting tumor cell proliferation. Their oral bioavailability and targeted approach make them convenient and effective. However, cross-reactivity with other kinases can lead to off-target effects and the emergence of resistance, necessitating the development of next-generation TKIs that can overcome these limitations.

ADCs, such as T-DM1 and T-DXd, combine the precision of mAbs with the potency of cytotoxic drugs, delivering a lethal payload directly to cancer cells. This approach minimizes the systemic toxicity associated with traditional chemotherapy. The success of ADCs such as T-DM1 in clinical trials has established them as a standard of care for certain patient populations. However, the complexity of their manufacturing and the potential for immune-related and off-target toxicity are areas that require ongoing research and optimization.

The field of HER2-targeted therapies is rapidly evolving, with numerous drugs in various stages of clinical development. For instance, T-DXd has shown promising results in phase III trials, such as the DESTINY-Breast03 trial, where it demonstrated superior efficacy compared to T-DM1 (Cortés et al., 2022). This has led to investigations into its use in the neoadjuvant setting, as seen in the DESTINY-Breast05 trial, which aims to assess its potential as an adjunct to intensive therapy in high-risk HER2+ EBC patients.

Apart from the development and selection of targeted drugs, additional strategies, including enhancing immune checkpoints and identifying biomarkers, are emerging in the HER2 field to pinpoint the ideal patient populations for novel therapeutic approaches. Overall, research on BC has overcome significant hurdles in recent decades with the advent of targeted therapies, yet continued in-depth research and the exploration of precision medicine are needed to make greater progress in this field (Emens et al., 2020).

## 7 Conclusion

Although significant progress has been made in the development of each class of HER2-targeted therapies, the optimal sequencing, combination, and individualization of these treatments remain critical areas of focus. Continued research is essential to refine our understanding of resistance mechanisms, to develop more effective and less toxic therapies and to identify biomarkers that can predict response to treatment. The future of HER2+ BC therapy holds promise with the potential for more personalized and effective treatment strategies.

## Figures and Tables

**Table:** 

**ADC**	antibody–drug conjugates
**ADCC**	antibody‒dependent cell‒mediated cytotoxicity
**AC-T**	anthracycline combined with cyclophosphamide followed by paclitaxel
**AC-TH**	anthracycline combined with cyclophosphamide followed by paclitaxel combined with trastuzumab
**AEs**	adverse events
**ATP**	adenosine triphosphate
**BBB**	blood‒brain barrier
**BM**	brain metastasis
**DC**	doxorubicin and cyclophosphamide
**DFS**	disease‒free survival
**TKI**	tyrosine kinase inhibitors
**VEGF**	vascular endothelial growth factor
**PTEN**	phosphatase and tensin homolog
**NK**	natural killer
**EGFR/HER1/ERBB1**	human epidermal growth factor receptor
**HR**	hormone receptor
**RTK**	receptor tyrosine kinases
**mAbs**	monoclonal antibodies
**MCL-2**	mantle cell lymphoma
**TRAIL**	tumor necrosis factor-related apoptosis-inducing ligand
**PI3K**	Phosphoinositide 3-kinase
**mTOR**	mammalian target of rapamycin
**NAT**	neoadjuvant therapy
**T-DM1**	trastuzumab emtansine
**FDA**	Food and Drug Administration
**T-DXd**	trastuzumab deruxtecan
**PFS**	progression‒free survival
**OS**	overall survival
**NACT**	neoadjuvant chemotherapy
**RCT**	randomized clinical trial
**PLD**	pegylated liposomal doxorubicin
**tpCR**	total pathologic complete response
**ORR**	objective response rate
**nab-PTX**	nanoparticle albumin-bound paclitaxel
**FEC**	fluorouracil and epirubicin plus cyclophosphamide
**THP**	paclitaxel and trastuzumab plus pertuzumab
**T-DM1+P**	trastuzumab emtansine plus pertuzumab
**TCbHP**	docetaxel, carboplatin, or trastuzumab plus pertuzumab
**TCbHy**	docetaxel, carboplatin, trastuzumab, and pyrotinib
**SABCS**	SAN Antonio Breast Cancer Seminar
**TCbH**	docetaxel, carboplatin combined with trastuzumab
**wTH**	weekly administration of paclitaxel and trastuzumab followed by trastuzumab every 3 weeks
**iDFS**	invasive disease-free survival
**non pCR**	patients who did not achieve a pathologic complete response
**NCCN**	National Comprehensive Cancer Network
**XIAP**	X is an inhibitor of apoptosis proteins
