# Supplementary material for: HER2-targeted therapies for HER2-positive early-stage breast cancer: present and future
Source: Front Pharmacol. 2024 Sep 16;15:1446414. doi: 10.3389/fphar.2024.1446414 (PMC11439691; doi:10.3389/fphar.2024.1446414)
Supplement: Supplementary file 1 [file Table1.DOCX]

Table S1. The sequence of amino acids used for the creation of trastuzumab and pertuzumab from DrugBank database.

|  | Light chain | Heavy chain |
| --- | --- | --- |
| Trastuzumab | DIQMTQSPSSLSASVGDRVTITCRASQDVNTAVAWYQQKPGKAPKLLIYSASFLYSGVPS  RFSGSRSGTDFTLTISSLQPEDFATYYCQQHYTTPPTFGQGTKVEIKRTVAAPSVFIFPP  SDEQLKSGTASVVCLLNNFYPREAKVQWKVDNALQSGNSQESVTEQDSKDSTYSLSSTLT  LSKADYEKHKVYACEVTHQGLSSPVTKSFNRGEC | EVQLVESGGGLVQPGGSLRLSCAASGFNIKDTYIHWVRQAPGKGLEWVARIYPTNGYTRY  ADSVKGRFTISADTSKNTAYLQMNSLRAEDTAVYYCSRWGGDGFYAMDYWGQGTLVTVSS  ASTKGPSVFPLAPSSKSTSGTAALGCLVKDYFPEPVTVSWNSGALTSGVHTFPAVLQSS  GLYSLSSVVTVPSSSLGTQTYICNVNHKPSNTKVDKKVEPKSCDKTHTCPCCPPCPELLGG  PSGVFLFPPKPKDTLMISRTPEVTCVVVDVSHEDPEVKFNWYVDGVEVHNAKTKPREEQYN  STYRVVSVLTVLHQDWLNGKEYKCKVSNKALPAPIEKTISKAKGQPREPQVYTLPPSREE  MTKNQVSLTCLVKGFYPSDIAVEWESNGQPEPNNYKTTPPVLDSGGSFFLYSKLTVDKSRW  QQGNVFSCSVMHEALHNHYTQKSLSLSPGK |
| Pertuzumab | DIQMTQSPSSLSASVGDRVTITCKASQDVSIGVAWYQQKPGKAPKLLIYSASYRYTGVPS  RFSGSGSGTDFTLTISSLQPEDFATYYCQQYYIYPYTFGQGTKVEIKRTVAAPSVFIFPP  SDEQLKSGTASVVCLLNNFYPREAKVQWKVDNALQSGNSQESVTEQDSKDSTYSLSSTLT  LSKADYEKHKVYACEVTHQGLSSPVTKSFNRGEC | EVQLVESGGGLVQPGGSLRLSCAASGFTFTDYTMDWVRQAPGKGLEWVADVNPNSGGSIY  NQRFKGRFTLSVDRSKNTLYLQMNSLRAEDTAVYYCARNLGPSFYFDYWGQGTLVTVSSA  STKGPSVFPLAPSSKSTSGGTAALGCLVKDYFPEPVTVSWNSGALTSGVHTFPAVLQSSG  LYSLSSVVTVPSSSLGTQTYICNVNHKPSNTKVDKKVEPKSCDKTHTCPPCPAPELLGGP  SVFLFPPKPKDTLMISRTPEVTCVVVDVSHEDPEVKFNWYVDGVEVHNAKTKPREEQYNS  TYRVVSVLTVLHQDWLNGKEYKCKVSNKALPAPIEKTISKAKGQPREPQVYTLPPSREEM  TKNQVSLTCLVKGFYPSDIAVEWESNGQPENNYKTTPPVLDSDGSFFLYSKLTVDKSRWQ  QGNVFSCSVMHEALHNHYTQKSLSLSPG |

Trastuzumab, commercial antibody: Herceptin, originally developed by Roche Pharma (Schweiz) Company Limited.

Pertuzumab, commercial antibody: Perjeta, produced by Genetic Engineering Technology Company.
