# Supplementary material for: HER2-targeted therapies for HER2-positive early-stage breast cancer: present and future
Source: Front Pharmacol. 2024 Sep 16;15:1446414. doi: 10.3389/fphar.2024.1446414 (PMC11439691; doi:10.3389/fphar.2024.1446414)
Supplement: Supplementary file 2 [file Table2.DOCX]

Table S2. Clinical trials of neoadjuvant therapy for HER2-positive early-stage breast cancer.

| Clinical trial | ClinicalTrials.gov  number | Total number of patients | Trial arms | Primary endpoint | Result |
| --- | --- | --- | --- | --- | --- |
| NSABP-B27(Abdel-Razeq et al., 2018) | NCT02001506 | 121 | four cycles of adriamycin and cyclophosphamide followed by four cycles of docetaxel with concomitant trastuzumab | DFS; OS | 3- and 5-year DFS: 84.2 and 74.1%, respectively; 3- and 5-year OS: 87.2 and 83.4%, respectively |
| Phase II and Biomarker Study(Wang et al., 2022) | NA | 54 | six cycles (every 3 weeks per cycle) of PLD plus docetaxel and trastuzumab intravenously for neoadjuvant treatment | tpCR | 48.0% |
| The TECHNO trial(Untch et al., 2011) | NCT00795899 | 217 | four 3-week cycles of epirubicin plus cyclophosphamide followed by four 3-week cycles of paclitaxel plus trastuzumab before surgery. Complete treatment with trastuzumab was continued 1 year after surgery. | DFS | 3-year DFS (88% vs. 73%) |
| Multicenter Phase II Trial(Tokunaga et al., 2019) | NA | 30 | four cycles nab-paclitaxel with trastuzumab every 3 weeks followed by four cycles of FEC | pCR | 74.0% |
| KRISTINE trial(Hurvitz et al., 2018) | NCT02131064 | 444 | docetaxel plus carboplatin plus trastuzumab plus pertuzumab vs. T-DM1 plus pertuzumab | pCR | 55·7% vs. 44·4% |
| NeoSphere trial(Gianni et al., 2012) | NCT00545688 | 417 | four cycles of trastuzumab plus docetaxel or pertuzumab and trastuzumab plus docetaxel (group B) or pertuzumab and trastuzumab (group C) or pertuzumab plus docetaxel (group D) | pCR | 29.0% vs. 45.8% vs. 16.8% vs. 24.0% |
| WSG-ADAPT trial(Nitz et al., 2017) | NCT01779206 | 160 | pertuzumab plus trastuzumab (TP) for four cycles ± weekly paclitaxel | pCR | 90.5% vs. 36.3% |
| PHEDRA trial(Wu et al., 2022) | NCT03588091 | 355 | four cycles of oral pyrotinib or placeboonce daily, plus intravenous trastuzumab and docetaxel every 3 weeks | tpCR | 41.0% vs. 22.0% |
| NeoATP trial(Yin et al., 2022) | NA | 53 | pyrotinib and trastuzumab with weekly paclitaxel-cisplatin for four cycles | pCR | For women with hormone receptor-negative and -positive tumors, the pCR rates were 85.71% and 59.38% |
| NeoALTTO(Baselga et al., 2012) | NCT00553358 | 455 | lapatinib and intravenous trastuzumab, or trastuzumab | pCR | 51·3% vs. 29·5% |
| Neo-LaTH(Tokunaga et al., 2021) | NA | 212 | neoadjuvant induction anti-HER2 therapy with lapatinib and trastuzumab followed by anti-HER2 therapy plus weekly paclitaxel with or without prolongation of anti-HER2 therapy | DFS; OS | 5-year DFS: 87.8%; 5-year OS: 95.6% |
| APTneo Michelangelo(Gianni et al.) | NCT03595592 | 661 | addition of atezolizumab (tecentriq) to neoadjuvant trastuzumab plus pertuzumab | pCR | 57.8% vs. 52.0% |

Abbreviations: HER2: human epidermal growth factor receptor 2; DFS: disease-free survival; OS: overall survival; pCR: athologic complete response; tpCR: total pathologic complete response; Pac: paclitaxel; HR: hazard ratio; CI: confidence interval; NAT: neoadjuvant therapy; HP: trastuzumab plus pertuzumab; T-DM1: trastuzumab emtansine.
