# Supplementary material for: HER2-targeted therapies for HER2-positive early-stage breast cancer: present and future
Source: Front Pharmacol. 2024 Sep 16;15:1446414. doi: 10.3389/fphar.2024.1446414 (PMC11439691; doi:10.3389/fphar.2024.1446414)
Supplement: Supplementary file 3 [file Table3.DOCX]

Table S3. Clinical trials of adjuvant therapy for HER2-positive early-stage breast cancer.

| Clinical trial | ClinicalTrials.gov  number | Total number of patients | Trial arms | Primary endpoint | Result |
| --- | --- | --- | --- | --- | --- |
| NCCTG N9831 trial(Perez et al., 2011) | NCT00005970 | 2184 | Doxorubicin plus cyclophosphamide, followed by paclitaxel (A); doxorubicin plus cyclophosphamide, followed by paclitaxel, followed by trastuzumab (B); doxorubicin plus cyclophosphamide, followed by paclitaxel plus trastuzumab (C) | DFS; OS | 5-year DFS rates of A and B are71.8% and 80.1%, respectively; 5-year DFS rates of B and C are 80.1% and 84.4% respectively |
| NSABP B-31/NCCTG N983 trial(Perez et al., 2014; Chumsri et al., 2019) | NA | 4066 | Doxorubicin plus cyclophosphamide followed by paclitaxel; doxorubicin plus cyclophosphamide, followed by paclitaxel plus trastuzumab, followed by trastuzumab | DFS; OS | adding trastuzumab led to a 37% relative improvement in OS and an increase in 10-year OS rate from 75.2% to 84%; an improvement in DFS of 40% and increase in 10-year DFS rate from 62.2% to 73.7% |
| HERA trial(Cameron et al., 2017) | NCT00045032 | 5099 | trastuzumab for 1 year or for 2 years or to the observation group | DFS | trastuzumab significantly reduced the risk of 1-year DFS (HR=0·76, 95% CI 0·68-0·86) and death (HR=0·74, 0·64-0·86) |
| BCIRG-006 trial(Slamon et al., 2011) | NCT00021255 | 3222 | doxorubicin or cyclophosphamide followed by docetaxel every 3 weeks (AC-T), the same regimen plus 52 weeks of trastuzumab (AC-T plus trastuzumab), or docetaxel and carboplatin plus 52 weeks of trastuzumab (TCH) | DFS | 5-year DFS rates were 75% (AC-T), 84% (AC-T), and 81% (TCH) |
| ExteNET trial(Chan et al., 2016) | NCT00878709 | 2840 | Docetaxel (12 months); docetaxel (12 months) followed by neratinib or placebo | iDFS | 2-year iDFS rate was 93·9% vs. 91·6% |
| KATHERIN trial(von Minckwitz et al., 2019) | NCT01772472 | 1484 | Trastuzumab given for 14 cycles after surgery every 3 weeks vs T-DM1 given for 14 cycles after surgery every 3 weeks | iDFS | 3-year iDFS was 77.0% vs. 88.3% |
| KATHERINE trial(Huang et al., 2021) | NA | 101 | 14 cycles of adjuvant T-DM1 or trastuzumab after taxane- and trastuzumab-containing neoadjuvant chemotherapy followed by surgery | iDFS | T-DM1 treatment resulted in a 43% reduction in risk of iDFS event compared to trastuzumab |
| DESTINY-Breast05 trial | NCT04622319 | NA | T-DXd vs.T-DM1 | iDFS | NA |
| APHINITY trial(Piccart et al., 2021) | NCT01358877 | 4805 | either 1-year pertuzumab or placebo added to standard adjuvant chemotherapy and 1-year trastuzumab | iDFS | pertuzumab added to adjuvant trastuzumab and chemotherapy improved iDFS (HR=0.81,95% CI, 0.66-1.00, P = 0.045) |

Abbreviations: DFS: disease-free survival; OS: overall survival; iDFS: invasive disease-free survival; HR: hazard ratio; CI: confidence interval; T-DM1: trastuzumab emtansine; T-DXd: trastuzumab deruxtecan; AC-T: anthracycline combined with cyclophosphamide followed by paclitaxel; TCH: docetaxel, carboplatin combined with trastuzumab.
